# Is There a Balance in Oxidative-Antioxidant Status in Blood Serum of Patients with Advanced Endometriosis?

**DOI:** 10.3390/antiox10071097

**Published:** 2021-07-08

**Authors:** Izabela Kokot, Agnieszka Piwowar, Marcin Jędryka, Ewa Maria Kratz

**Affiliations:** 1 Department of Laboratory Diagnostics, Division of Laboratory Diagnostics, Faculty of Pharmacy, Wroclaw Medical University, Borowska Street 211A, 50-556 Wroclaw, Poland; ewa.kratz@umed.wroc.pl; 2 Department of Toxicology, Faculty of Pharmacy, Wroclaw Medical University, Borowska Street 211, 50-556 Wroclaw, Poland; agnieszka.piwowar@umed.wroc.pl; 3 Department of Oncology, Gynecological Oncology Clinic, Faculty of Medicine, Wroclaw Medical University, Hirszfeld Square 12, 53-413 Wroclaw, Poland; marcin.jedryka@umed.wroc.pl; 4 Department of Oncological Gynecology, Wroclaw Comprehensive Cancer Center, Hirszfeld Square 12, 53-413 Wroclaw, Poland

**Keywords:** endometriosis, blood serum, oxidative-antioxidant balance, oxidative stress parameters, total antioxidant capacity

## Abstract

Can redox homeostasis indicators be potential non-invasive markers, crucial in the diagnosis and treatment of endometriosis? We checked if the differences in levels of serum oxidative-antioxidant balance parameters (TAS, FRAP, albumin, total bilirubin, uric acid, iron, SIRT3, SIRT5, SIRT6, telomerase, AOPP) are significant between patients with advanced endometriosis (E), healthy women (control group, C) and non-endometriosis women, but with other gynecological disorders (NE). The FRAP concentrations were significantly higher in E and NE group than in the control group (*p* = 0.015 and *p* = 0.017, respectively). The telomerase concentrations were significantly higher in the endometriosis group than in the control group (*p* = 0.004). Significantly higher concentrations of AOPP were observed in E (*p* < 0.001) and NE groups (*p* = 0.028) in comparison to the control subjects. Between stages III and IV of endometriosis, a significant difference existed only in concentration of iron (*p* = 0.013). There were no significant differences between the studied groups in the values of the remaining parameters. Based on the results of ROC curve analysis, we can conclude that the levels of serum FRAP, telomerase and AOPP may be taken into account as promising diagnostics markers that reflect the degree of oxidative stress accompanying advanced endometriosis.

## 1. Introduction

Endometriosis is one of the chronic benign gynecological disorders where endometrial tissue is present outside of the uterine cavity. Endometriotic lesions, observed mainly on the pelvic peritoneum, lead to a local inflammation associated with implantation, adhesions and proliferation of endometriotic cells. As result, especially in reproductive age women, it can cause a pelvic pain and result in infertility [1,2]. One of the possible factors influencing endometriosis development, besides inflammation, could be oxidative stress (OS). Oxidative stress is observed when the oxidative-antioxidant balance is disturbed [3,4]. The increase in the number of free radicals and/or the weakening of antioxidant systems causes the intensification of oxidative stress, which results in damage of circulating proteins, lipids, carbohydrates and nucleic acids [5]. Moreover, it is indicated that the oxidative stress present in endometriosis is not limited to the peritoneal cavity, but is rather a systemic condition [5]. This expansion of local inflammation and oxidative stress in endometriosis may result from the permeability properties of the peritoneal membrane [6,7]. Additionally, blood, more than the intracellular environment, is exposed to oxidants because it is a carrier of many biologically active substances. For this reason, as a part of the redox balance, it should contain a higher concentration of antioxidants than the inside of the cell, but this is not so certain [8,9]. However, due to the transport function of blood, it plays a key role in the distribution of antioxidants to every part of the body [10].

Serum concentrations of various antioxidants can be measured separately or summed, and given as the total antioxidant capacity (TAC) of the sample. The second idea appears to be more practical, because analyzing every single antioxidant is time consuming, labor intensive, costly and requires complex techniques [11]. There are numerous analytical methods for total antioxidant capacity determinations, and both methods used for concentration measurements of Trolox equivalent antioxidant capacity (TEAC; the method we used was called by the manufacturer as total antioxidant status, TAS) and ferric reducing antioxidant power (FRAP) belong to the analytical methods based on the single electron transfer (SET), in which the reducing capacity toward any molecule by electron donation is measured [12,13].

Very important parts of the body’s antioxidant defense are endogenous low-molecular antioxidants that interact with free radicals and oxidants, contributing to their inactivation [14]. This group includes, among others, albumin (ALB), total bilirubin (T-BIL) and uric acid (UA). In humans, albumin is the most abundant protein in the blood, which is synthesized in the liver, and its main functions are the regulation of oncotic pressure, binding and transport of endogenous and exogenous compounds as well as antioxidant activity [8,9,15]. Bilirubin is constantly produced in the human body as it is a product of heme breakdown. It shows antioxidant properties as a scavenger of selected free radicals. Uric acid, which is the end product of purine metabolism, has also strong scavenging properties against oxidants [14].

Considering the complex environment of the peritoneal cavity, it is hard to find the major source of oxidative stress that may occur in relation to endometriosis [5]. In the place where endometriotic lesions are, it comes to erythrocytes hemolysis and accumulation of iron which induce oxidative stress [16]. Fenton’s reaction is of key importance in that induction, during which reactive oxygen species (ROS) are produced, influencing the intensity of oxidative stress. In this reaction, iron can act as a catalyst, generating one of the strongest reactive oxygen species—the hydroxyl radical. In the human body, iron is released from hemoglobin and heme by the action of macrophages [5,17]. Additionally, in retrograde menstruation process, the apoptotic endometrial tissue and desquamated menstrual cells are transported into the peritoneal cavity, which can promote a chronic inflammation with cytokine production and induction of immune cells, such as granulocytes and macrophages. When these cells are activated, they produce a large amount of reactive oxygen species, which increases oxidative stress and inflammation [18,19]. These harmful reactions can be prevented or inhibited by antioxidants, whose definition is “any substance that, when present at low concentrations compared to those of an oxidisable substrate, significantly delays or prevents oxidation of that substrate” [20].

Recently, there has been an increase in the number of study results confirming the significant role of sirtuins (SIRT) in the regulation of cell homeostasis (reviewed in: [21,22,23]). Sirtuins are of particular importance in the regulation of cellular metabolism, including that associated with chronic inflammation—responsible for the generation and maintenance of oxidative stress [21,24]. Sirtuins have diverse cellular localization—SIRT3 and SIRT5 are mitochondrial sirtuins, whereas SIRT6 is present predominately in the nucleus, and a large fraction of SIRT6 is associated with heterochromatin. These sirtuins have also different activities: SIRT3 has both deacetylase and mono-ADP-ribosyl transferase activities, SIRT5 reveals weak deacetylase activity and SIRT6 is mono-ADP-ribosyl transferase [21,22,25]. SIRT3 affects key mitochondrial functions such as ATP production, regulation of reactive oxygen species generation, β-oxidation, cell death and ketogenesis [21,26]. SIRT5 possesses high desuccinylation, demalonylation and deglutarylation activities. It activates the urea cycle by deacetylating carbamoyl phosphate synthetase 1 [27].

Telomerase (TE) is an enzyme, a ribonucleoprotein complex consisting of two main components, the RNA-part containing the template region for telomere synthesis and the catalytic telomerase reverse transcriptase. The fundamental function of telomerase is the maintenance of the telomeres that cap the ends of chromosomes, thereby regulating the proliferative lifespan of a cell. In addition to maintaining telomere length, telomerase may also have a role in proliferating and modulating the expression of growth-promoting genes [28].

In the human blood plasma, proteins are in a relatively high concentration with capacity to scavenge some reactive species generated during oxidative stress. This makes advanced oxidation protein products (AOPPs) a possible marker of oxidative damage. The plasma level of AOPPs rises with age, but also in chronic diseases, such as endometriosis [29,30]. AOPPs are a group of dityrosine-, pentosidine- and carbonyl-containing protein products generated by reaction of plasma proteins with hypochlorous acid (HOCl) and chloramines during oxidative stress, and AOPPs are carried mainly by albumin in the blood circulation [30]. Additionally, AOPPs are factors in the response to oxidative stress in vivo by activating the oxidative metabolism of neutrophils and monocytes, and circulate in the blood of patients for a long time because their degradation by cells takes hours or even days [31].

The assumptions of our work follow from the growing need for new therapeutic targets and biomarkers for the non-invasive diagnosis of endometriosis. The primary goal of our study was to determine the ability of each of the analyzed parameters (TAS, FRAP, albumin, total bilirubin, uric acid, iron, SIRT3, SIRT5, SIRT6, telomerase and AOPP) to differentiate patients with advanced endometriosis from the group of healthy women and the group of women without endometriosis (non-endometriosis), but with other benign gynecological disorders. Additionally, we were interested if the values of the examined markers may differentiate stage III from stage IV of endometriosis. The next aim of our study was to check the associations between examined serum parameters of oxidative-antioxidant balance, also in relation to the previously determined parameters of inflammation.

## 2. Materials and Methods

### 2.1. Patients

Serum samples were collected from 43 women with endometriosis (E), 35 women without endometriosis as comparative group (NE) and 18 healthy female volunteers as the control group (C). All participants in the study were of similar age and had a comparable body mass index (BMI). Two first groups of patients (E and NE) were recruited and collected at the Department of Gynecological Oncology of Wroclaw Comprehensive Cancer Center (Poland). These groups underwent surgical interventions and, after histological verification, were assigned to the proper group. The group of women with endometriosis was also classified based on the extent and severity of the disease, according to the American Fertility Society revised (rAFS) classification. All patients from the endometriosis group had confirmed advanced stages of the disease, where 20 women were distinguished with a moderate (III E) and 23 women with a severe (IV E) stage of endometriosis. The NE group of patients was histologically confirmed as other-than-endometriosis benign disorders, e.g., leiomyomas, benign ovarian cyst or with severe dysplasia—CIN 3 (cervical intraepithelial neoplasia grade 3). The control group consisted of volunteers—healthy, non-pregnant, premenopausal women, without any gynecological problems and symptoms of illness, with no disease history connected with endometriosis, who were recruited from among employees of the Wroclaw Medical University and from our friends. The basic characteristics of the analyzed groups are presented in Table 1.

All of the information on blood collection and handling is described in our previous work [32]. The study was conducted in agreement with the Helsinki-II declaration, and the protocol was approved by the Bioethics Human Research Committee of the Wroclaw Medical University (Permission No. KB-231/2019 and KB-685/2019). All participants gave their written consent prior to the study.

### 2.2. Total Antioxidant Capacity Concentration

Total antioxidant capacity was measured as total antioxidant status—TAS and as ferric reducing antioxidant power—FRAP. TAS was determined automatically on biochemical analyzer Konelab 20i^®^ (ThermoScientific, Vantaa, Finland), using the colorimetric method (Randox TAS Kit, Crumlin, United Kingdom) with linearity of up to 2.50 mmol/L. The results were expressed in mmol/L of Trolox equivalents because a calibration curve was constructed for this standard. The ferric reducing antioxidant power was measured using the colorimetric method with ferric tripyridyltriazine [33]. FRAP reagent was prepared *ex tempore* by mixing 300 mM acetate buffer (pH 3.6), 10 mM 2,4,6-tripyridyl-s-triazine (TPTZ) in 40 mM HCl and 20 mM aqueous solution of FeCl_3_ × 6H_2_O in proportion 10:1:1, respectively. 500 μL of FRAP reagent was mixed with 100 µL of a 1:9 diluted sample, incubated for 5 min at 37 °C and then centrifuged at 2000× *g* for 10 min at room temperature. The supernatants were analyzed spectrophotometrically at 593 nm against a reagent blank using a UV/Vis spectrophotometer (UV-6300PC, VWR, Shanghai, China). A calibration curve was performed for the known amounts of Fe^2+^ in the solution, from 0.05 to 0.25 mM Fe^2+^.

### 2.3. Low-Molecular-Weight Antioxidants and Iron Measurements

Concentrations of albumin, total bilirubin, uric acid and iron were measured by the colorimetric method using biochemical analyzer Konelab 20i^®^ (ThermoScientific, Vantaa, Finland). All determinations were carried out in accordance with the manufacturer’s instructions. Lower test limits were: 2.00 g/dL, 0.06 mg/dL, 0.20 mg/dL and 6.00 μg/dL, respectively.

### 2.4. Sirtuins and Telomerase Concentrations

The concentrations of SIRT3, SIRT5, SIRT6 and TE were measured with commercially available ELISA tests, according to the recommendations of the manufacturer. Sirtuin concentrations were examined with Human Sirtuin 3 ELISA Kit, Human Sirtuin 5 ELISA Kit and Human Sirtuin 6 ELISA Kit (Bioassay Technology Laboratory, Shanghai, China), and human telomerase concentrations were measured with Human Telomerase (TE) ELISA Kit (CUSABIO Technology LLC, Wuhan, China). To determine the concentrations of the above parameters, Mindray-96A reader (Mindray, Shenzhen, China) was used. Lower standard curve ranges were: 0.10 ng/mL, 0.10 ng/mL, 0.10 ng/mL and 0.312 ng/mL, respectively.

### 2.5. Oxidative Protein Damage Measurements

The concentration of advanced protein oxidation products (AOPPs) were measured according to the method of Witko-Sarsat et al. [34], where AOPP reacts with potassium iodide solution in an acidic environment. In the first step, 20 μL of KI was added to 400 μL of serum samples, diluted previously 5-fold with PBS. After 2 min incubation at room temperature, 40 μL of 10% (*v*/*v*) acetic acid was added, mixed thoroughly and immediately measured at 340 nm. The results were expressed in μmol/L of chloramine T equivalent because a calibration curve was constructed for chloramine T concentrations ranging from 0 to 100 μM. The concentration of advanced protein oxidation products were analyzed using a UV/V is spectrophotometer (UV-6300PC, VWR, Shanghai, China).

### 2.6. Statistical Analysis

Statistical analysis was performed with the Statistica 13.3 PL software (StatSoft Poland Sp. z o.o., Krakow, Poland). All data were tested with the Shapiro–Wilks test for normality determination. Statistical significance of all the values was determined with the Mann–Whitney U test for comparison of the data obtained for two groups, and Kruskal–Wallis tests for multiple-group comparison. Using the Spearman rank test, the correlations between the analyzed parameters were checked, and a *p* value of less than 0.05 was considered as significant. The results were presented as mean ± SD (SD—standard deviation) and as median with interquartile range (Q1–Q3) on the graphs. The clinical value of the determined oxidative-antioxidant balance parameters was analyzed using receiver operating characteristic (ROC) curves. In the following step, univariate and multivariate logistic regressions were performed. Additionally, we correlated the analyzed oxidative-antioxidant balance parameters with the values of inflammatory parameters previously determined by us [32] (Supplementary Table S3).

## 3. Results

**Figure 1:**
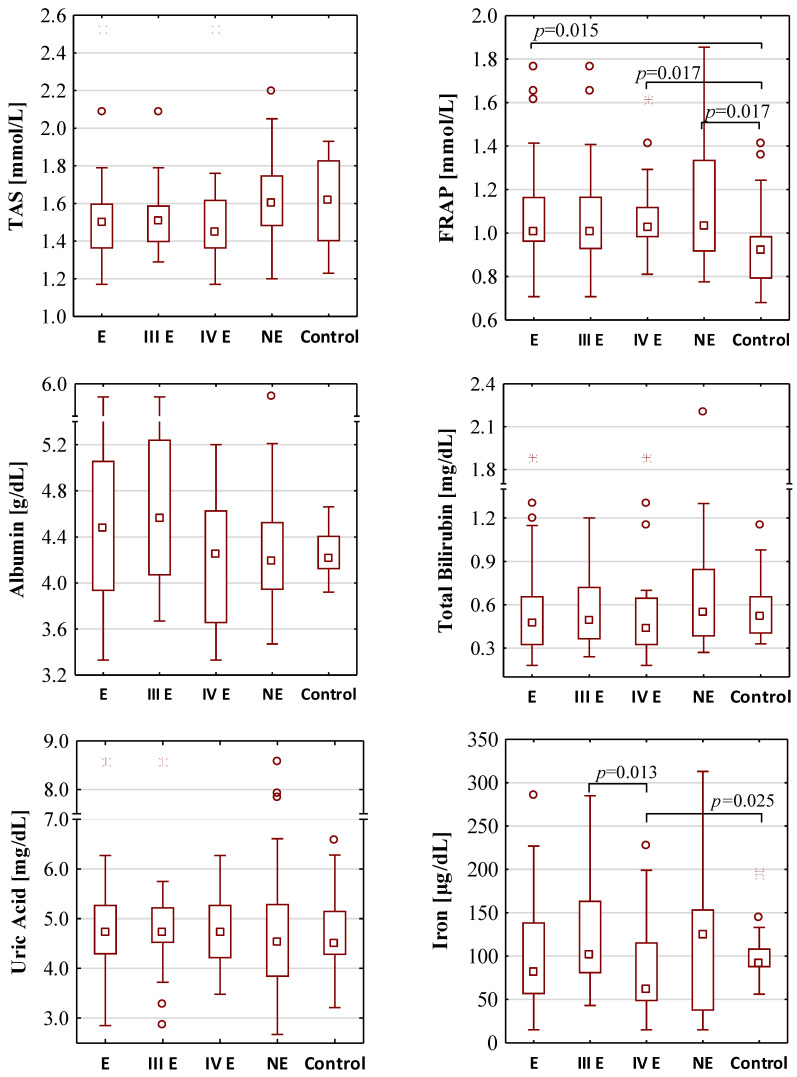

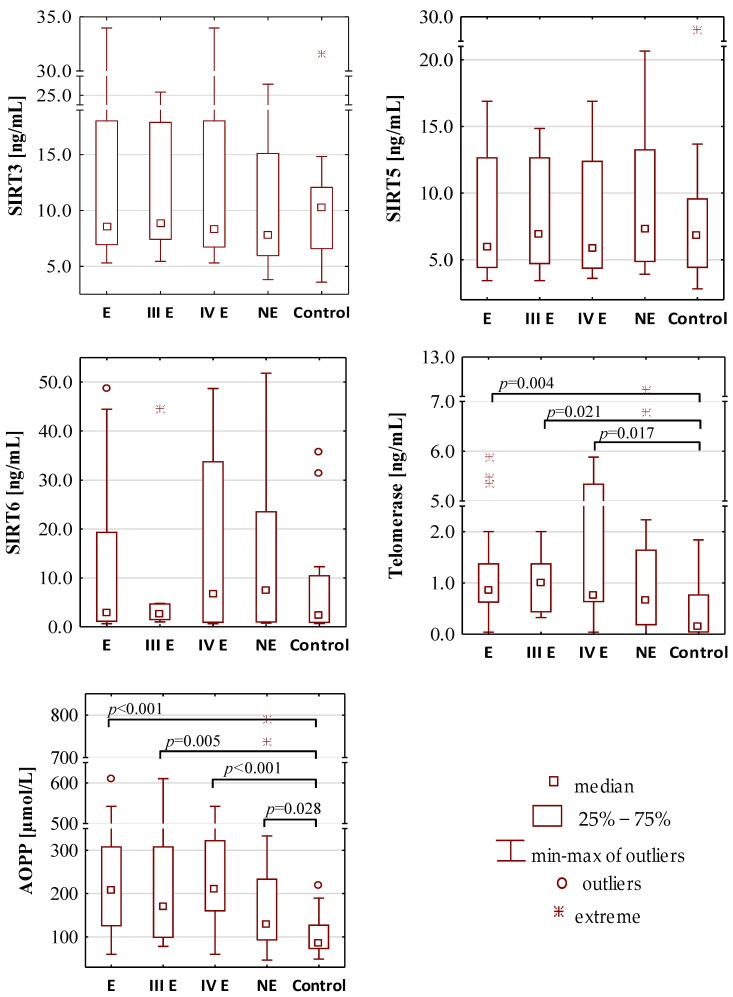
The concentration values of oxidative-antioxidant balance parameters. E—endometriosis; NE—non-endometriosis groups. Control—group of healthy volunteers; III E and IV E—III and IV stage of endometriosis, respectively. A two-tailed *p*-value of less than 0.05 was considered significant. AOPP—advanced protein oxidation products; FRAP—ferric reducing antioxidant power; SIRT3—sirtuin 3; SIRT5—sirtuin 5; SIRT6—sirtuin 6; TAS—total antioxidant status.

The results of the determination of TAS, FRAP, albumin, total bilirubin, uric acid, iron, SIRT3, SIRT5, SIRT6, telomerase and AOPP concentrations are shown in Table 2 and Figure 1. The significant correlations between concentration values of investigated parameters, for all studied subjects, are shown in Table 3. The results of univariate and multivariate logistic regression models are summarized in Supplementary Materials (Supplementary Tables S1 and S2, respectively).

### 3.1. The Concentrations of Total Antioxidant Capacity

The concentration values of total antioxidant status were similar in each group. The median values of TAS for the endometriosis, the non-endometriosis and the control group were 1.50 mmol/L, 1.60 mmol/L and 1.62 mmol/L, respectively. There were also no differences between TAS concentrations between groups of moderate (III) and severe (IV) stage of endometriosis (median values: 1.51 mmol/L and 1.45 mmol/L, respectively). FRAP concentrations were significantly higher in the endometriosis and non-endometriosis group than in the control group (median values: 1.01 mmol/L, 1.03 mmol/L and 0.92 mmol/L, respectively) with the significance of *p* = 0.015 and *p* = 0.017, respectively. FRAP concentrations were also significantly higher (*p* = 0.017) in serum samples of patients with severe endometriosis (median value 1.02 mmol/L) when compared with the control group, while such a difference was not observed for stage III of the disease (median value 1.00 mmol/L). Women with stage III and IV of endometriosis have similar serum FRAP concentrations.

### 3.2. The Concentrations of Low-Molecular-Weight Antioxidants and Iron

No significant differences were found in the concentrations of serum albumin, total bilirubin, uric acid and iron between the endometriosis (median values: 4.48 g/dL, 0.47 mg/dL, 4.72 mg/dL and 82.00 μg/dL, respectively), non-endometriosis (median values: 4.18 g/dL, 0.54 mg/dL, 4.53 mg/dL, and 124.00 μg/dL, respectively) and control (median values: 4.22 g/dL, 0.52 mg/dL, 4.50 mg/dL and 91.00 μg/dL, respectively) groups. Between stages III and IV of endometriosis, significant differences in concentrations of iron were observed (median values: 101.50 μg/dL and 62.00 μg/dL, *p* = 0.013); additionally, the concentrations of iron were significantly lower (*p* = 0.025) in sera of patients in IV stage of endometriosis than in the control group.

### 3.3. The Concentrations of Sirtuins and Telomerase

The serum concentrations of SIRT3, SIRT5 and SIRT6 were similar in the endometriosis (median values: 8.53 ng/mL, 5.96 ng/mL and 2.94 ng/mL, respectively), non-endometriosis (median values: 7.85 ng/mL, 7.30 ng/mL and 7.38 ng/mL, respectively) and control group (median values: 10.28 ng/mL, 6.80 ng/mL and 2.20 ng/mL, respectively). These parameters were also on a comparable level in stage III (median values: 8.85 ng/mL, 6.89 ng/mL and 2.66 ng/mL, respectively) and stage IV (median values: 8.34 ng/mL, 5.83 ng/mL and 6.64 ng/mL, respectively) of endometriosis. The telomerase concentration was significantly higher in the endometriosis group (median value: 0.86 ng/mL) than in the control group (median value: 0.15 ng/mL, *p* = 0.004). Significantly higher telomerase concentrations were also observed in the serum samples of patients with moderate (median value: 1.01 ng/mL, *p* = 0.021) and severe (median value: 0.75 ng/mL, *p* = 0.017) endometriosis group in comparison to the healthy women from the control group.

### 3.4. The Concentrations of Advanced Protein Oxidation Products

Significantly higher concentrations of AOPP were observed in the sera of patients with endometriosis (median value: 208.10 µmol/L, *p* < 0.001) and non-endometriosis group (median value: 129.58 µmol/L, *p* = 0.028) in comparison to the control subjects (median value: 83.92 µmol/L). The analysis of AOPP values showed that, in the moderate stage (median value: 168.31 µmol/L, *p* = 0.005) and the severe stage (median value: 209.86 µmol/L, *p* < 0.001) of endometriosis, they were significantly higher than in the control group.

### 3.5. ROC Curve Analysis

**Figure 2:**
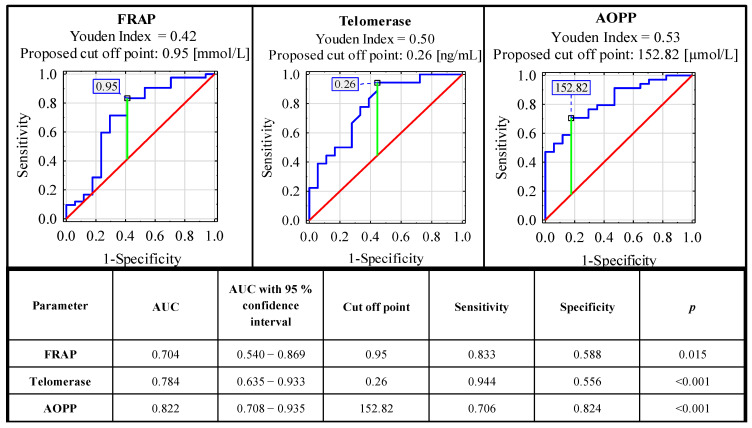
ROC curves for serum concentrations of FRAP, AOPP and telomerase as potential markers of advanced endometriosis. Data are given as AUC with 95% confidence interval. A *p* value of less than 0.05 was considered statistically significant. AOPP—advanced protein oxidation products; FRAP—ferric reducing antioxidant power. For AUC in the range of values 0.7–0.9, the clinical value is moderate.

ROC curve analysis carried out for concentrations of serum oxidative-antioxidant balance parameters in the endometriosis and the control group identified parameters with significant clinical value. From all the analyzed parameters, only FRAP, telomerase and AOPP could be considered as markers useful in endometriosis diagnostics. The sensitivity and specificity of these parameters were, respectively: 0.833, 0.588 (AUC 0.704—moderate clinical value); 0.944, 0.556 (AUC 0.784—moderate clinical value); and 0.706, 0.824 (AUC 0.822—moderate clinical value). For the determination of cut-off points, the Youden index method was used (Figure 2). The clinical value of a laboratory test with AUC can be defined as: 0–0.5—zero, 0.5–0.7—limited, 0.7–0.9—moderate and >0.9 high [35].

## 4. Discussion

Oxidative stress can be assessed from a pro-oxidative and/or anti-oxidative perspective. In both aspects, the examination of these processes may be presented as a whole, or as a value of individual component. This total effect of antioxidant capacity seems to be an appropriate measure of the simultaneous assessment of endogenous and exogenous antioxidants as a response to oxidative stress. The determined concentration values of serum TAS were slightly and insignificantly lower in the endometriosis group than in the non-endometriosis and control groups (mean values: 1.52 mmol/L, 1.61 mmol/L and 1.60 mmol/L, respectively). Our results correspond to the results obtained by Ferreira et al. [36], where the values of total antioxidant capacity (same method as our TAS) were lower in sera of infertile women with endometriosis than in those without disease, but also insignificantly so. However, patients examined by the authors were at stage I or II of endometriosis, whereas, in our study, endometriosis women represented advanced stages (stages III and IV). On the other hand, as observed by Turgut et al. [37], lower TAS values in sera of women with advanced stage of endometriosis, in comparison to the control group of healthy women, were significant (*p* < 0.001, 1.01 mmol/L and 1.15 mmol/L, respectively). In addition, Jana et al. [38] observed a noteworthy lower total antioxidant capacity in sera of women with endometriosis in comparison to the control group (733.6 µmol/L vs. 936.3 µmol/L, *p* ≤ 0.001, respectively). The authors [38] used the same standard as we did (Trolox) in the preparation of calibration curve, but our TAS values slightly differ, although they are comparable. Most likely, it was because of methodological differences; in the case of Jana et al. [38], the chemiluminescence method was applied. However, it is worth noting that the correlation between the chemiluminescence method and that used by us (the colorimetric method) has been documented based on the formation of ABTS+ radical ions [39]. Another explanation of the lack of differences in TAS values between the groups examined by us may be the lower number of subjects in each group in the present study, in contrast to the more numerous groups analyzed in the study of Jana et al. [38], which is important in statistical analyses.

The second parameter describing the total antioxidant capacity is ferric reducing antioxidant power. We observed a presence of strong positive correlations between TAS and FRAP outcomes (r = 0.576, *p* < 0.001), but there are conflicting data on this in the literature. Jansen and Ruskovska [40] also reported the presence of a positive, very high correlation between the above-mentioned methods (r = 0.807, *p* < 0.005); however, in some studies, no correlation has been demonstrated [41]. Moreover, FRAP values were lower than TAS (Table 2) and, in opposition to TAS, we showed that the FRAP concentrations were significantly higher in the endometriosis and non-endometriosis group than in the group of healthy women (mean values: 1.08 mmol/L, *p* = 0.015; 1.14 mmol/L, *p* = 0.017; and 0.95 mmol/L, respectively). The significant differences in FRAP levels between E and the control group, as observed by us, are most probably caused by stage IV of endometriosis, not by stage III, because only between stage IV of endometriosis and the control group do the significant differences in FRAP values exist (*p* = 0.017). The primary reason of FRAP levels’ underestimation in serum, in comparison to TAS, is that the FRAP method cannot detect compounds that act by radical quenching (H transfer), particularly thiols and proteins [42]. The methods used for the determination of total antioxidant capacity differ in the aspect of the components measured. The main known components influencing serum TAS levels are albumin and uric acid (28.0% and 19.3%, respectively), whereas, for serum FRAP, they are uric acid and ascorbic acid (61.7% and 10.1%, respectively) [41]. The above relationships were confirmed by the study of Benzie and Strain [33], who showed very strong positive correlations between concentrations of blood plasma FRAP and uric acid (r = 0.914, *p* < 0.001), and suggested that UA may play an important role in antioxidant defense, taking part in recycling of antioxidants [33]. We also showed a presence of high positive correlation between FRAP and UA concentrations (r = 0.590, *p* < 0.001). The variability of components measured by TAC’s methods is caused by differences in clinical situations related to the induction of oxidative stress [43]. Uric acid very strongly influences FRAP levels, and the significance of the measured FRAP value depends on UA concentrations. The serum concentration of UA may be increased or decreased due to the clinical condition and is also gender-dependent. In turn, the quantitative changes in the levels of total protein are reflected in the concentration of TAS [40,41,43].

TAS and FRAP are methods reflecting total antioxidant capacity, and despite the observed correlations, the results differ from each other and may lead to misleading conclusions. Therefore, when assessing the oxidative-antioxidant balance, it is advisable to use several parameters that differ in terms of methodology [40], and a careful analysis of all potential components should be the basis for the selection of an appropriate TAC method for adequate interpretation. However, it should be noted that the human body has a very effective antioxidant defense system that constantly controls oxidative stress generated by free radicals and other reactive compounds produced as an integral part of human metabolism. The stability of this system was confirmed by the study of Cao and Prior [41], who verified the variability of FRAP and TEAC (our TAS) in blood serum over 8 weeks, and documented that the concentration values of these parameters did not change with time.

Albumin is one of the main components of the antioxidant defense, both under normal and under oxidative stress conditions [44]. In the present study, the albumin concentrations were slightly higher, but insignificant, in endometriosis, when compared with the non-endometriosis and control groups (mean values: 4.45 g/dL, 4.29 g/dL and 4.26 g/dL, respectively). Hasan et al. [45] reported the similar values of albumin concentration, with no significant differences between the endometriosis and control groups (mean values: 4.42 g/dL and 4.62 g/dL, respectively). Albumin is also known as an effective free radical scavenger [46] and has the major input to plasma sulfhydryl groups, which can work as a chain-breaking antioxidant by donating an electron to neutralize a free radical [11]. Under physiological conditions, albumin does not carry iron, but in iron overload, it can bind Fe (II) and Fe (III). By binding iron and copper cations, albumin significantly reduces their activity: the bound ions are still available for reaction, but the free radicals formed immediately attack the albumin molecule itself and do not interact with other blood components. In this case, the albumin molecule is damaged; however, due to the high concentration of the protein, the damage is biologically insignificant [47,48]. Moreover, bilirubin bound by albumin is also an effective radical scavenger, and it has been suggested that it may play a particularly critical role in protection from oxidative damage process [11]. Benzie and Strain [33] reported that 5% of relative contributions to the FRAP level is for bilirubin. However, in our study, total bilirubin levels were similar in all of the studied groups.

Uric acid is known as a direct free radical scavenger, and part of its antioxidant activity can be associated with the formation of stable non-reactive iron complexes [11]. We did not observe the presence of significant differences between the analyzed groups of women; however, on the other hand, uric acid concentration positively correlated with TAS and FRAP concentrations (r = 0.264, *p* = 0.002 and r = 0.590, *p* < 0.001, respectively). It may be an additional proof that it is the main component of the antioxidant system, particularly that uric acid can be relevant in protection against some oxidizing agents such as ozone [11].

The serum iron concentrations were similar between the E, NE and control groups; however, when we separately checked the endometriosis group based on rAFS classification, we observed that in the group of women with severe endometriosis (stage IV), iron concentration was significantly lower (*p* = 0.013) than in the group of patients with stage III (median values: 62.00 μg/dL and 101.50 μg/dL, respectively). Additionally, the concentration of iron was significantly lower in serum of patients in stage IV of endometriosis than in the control group (median value: 91.00 μg/dL, *p* = 0.025). Similar to our results of iron concentrations, these were also reported by Chmaj-Wierzchowska et al. [49], where the mean value was 87.20 µg/dL in the blood sera of patients with endometrial cysts. The authors found no differences in iron levels when endometriosis women were compared to the group of patients with teratoma (mean: 78.01 µg/dL), which can be regarded as similar to our non-endometriosis group. On the contrary, Alizadeh et al. [17] showed significantly higher values of serum iron in patients with endometriosis than in the control group; however, the authors did not provide information on the stage of the disease: what, according to our observations, seems to be of key importance, as we have noticed the differences in iron levels between stages III and IV of the disease. Iron levels are likely to be highest in stage I of endometriosis and decline with the severity of the disease, which could be indicated by the fact that the iron levels we determined were noticeably higher in stage III than in stage IV; although these differences were not significant. Nevertheless, this hypothesis should be confirmed by conducting investigations also on groups of patients with endometriosis in stage I and II. Alizadeh et al. [17], in the ROC curve analysis for serum iron, showed a highly significant discriminant ability with an AUC value of 0.899 with a cut-off point of 173.3 µg/dL. The authors suggested that iron, as an important pro-oxidant factor, may be used as a good factor to distinguish the endometriosis patients from healthy subjects, but we did not observe a significant discriminant value for serum iron. Apart from the fact that the authors do not provide the stage of endometriosis in their study group, which makes it difficult to compare the results obtained by us to their results, it should be also emphasized that serum iron levels fluctuate daily, which is also observed during the menstrual cycle. Low iron levels are associated with anemia, as well as with acute and chronic infections and inflammatory reactions [49]. For the confirmation of above, we have found a significant negative correlation between serum iron concentrations and concentrations of hs-CRP and IL-6 (r = −0.540, *p* < 0.001 and r = −0.438, *p* < 0.001, respectively), which were examined by us previously [32] (Supplementary Materials, Table S3).

Sirtuins, a NAD^+^-dependent deacetylases, among their various functions, may also respond to oxidative stress [21,22,50]. Shi et al. [51] have reported that prolonged expression of SIRT3 decreases mitochondrial membrane potential and ROS production, while increasing cellular respiration. These studies concerned brown adipose tissue, but the authors suggest that SIRT3 may also exert a similar effect in other tissues in which it is expressed. It was also mentioned that induced SIRT3 activity is a result of reduced oxidative stress in mitochondria, which is particularly important because most of cellular oxidants are formed in the mitochondrial matrix [24]. SIRT5 desuccinylative and deglutarylative activity protect cells from oxidative damage by activating NADPH-producing enzymes. SIRT6 is involved in telomeres stabilization, DNA double strand break repair and regulation of transcription [27]. Sirtuins work together to respond to changes in the organism, including inflammatory factors and oxidative stress [21,22,50]. The requirement of NAD^+^ as an essential factor in sirtuin-catalyzed reactions suggests that they may be the kind of sensors for cellular energy and redox status linked to the metabolic state of the cell [25]. As far as we know, our study was the first analyzing serum sirtuin 3, 5 and 6 concentrations in endometriosis. Although we did not observe any significant differences in sirtuins concentrations between the analyzed groups, they were visibly higher in the endometriosis and non-endometriosis groups than in the control group. It should be also noticed that we observed very strong positive correlations between concentrations of SIRT3 and SIRT5 (r = 0.845, *p* < 0.001), SIRT3 and SIRT6 (r = 0.770, *p* < 0.001) as well as SIRT5 and SIRT6 (r = 0.863, *p* < 0.001), which additionally confirms that sirtuins are enzymes whose action depends on one another.

González-Fernández et al. [27], in the study of sirtuins’ gene expression profiles on human granulosa-lutein cells of infertile women, have observed only intermediate expression levels of SIRT3, SIRT5 and SIRT6 in endometriosis (advanced stages) compared with the control group of women with no ovarian factor. Related to clinical parameters, SIRT6 was correlated positively with FSH and LH doses administered in endometriosis patients [27]. One of the limitations of our study may be the collection of venous blood samples regardless of the day of the menstrual cycle. It has been documented that the assessment of the oxidative-antioxidant balance combined with the menstrual cycle phase may reflect the degree of disease activity: static, progressive or informing about relapse after therapy [3,5]. This can be important, especially in view of the fact that estrogens have a protective effect on oxidative damage [52]. On the other hand, not all studies support this protective function of hormones [53].

We have also shown very strong positive correlations between SIRT3, SIRT5 and SIRT6 with YKL-40 (r = 0.743, *p* < 0.001; r = 0.798, *p* < 0.001 and r = 0.829, *p* < 0.001, respectively)—Supplementary Materials, Table S3, and the elevated levels of YKL-40 in sera of women with endometriosis in comparison to the healthy subjects [32]. Among others, YKL-40 plays a key role in oxidant-induced injury responses and inflammation by regulating a number of basic biological processes, including oxidative injury, apoptosis or Th1/Th2 inflammatory balance [54]. The strong association of YKL-40 levels with the concentrations of sirtuins 3, 5 and 6 seems to be an interesting direction for further study, especially in the context of early stages of endometriosis, because, to the best of our knowledge, there are no such data available.

Oxidative stress is appeared to play an important role in the increased rate for shortening of telomeres. According to that observation, it is proposed that the levels of the enzyme telomerase might be associated with increased oxidative stress leading to higher telomerase levels [55]. In endometriosis development and progression, oxidative stress plays an important role, which was confirmed in the present study by the significantly higher serum telomerase concentrations in advanced endometriosis, compared with the control group of healthy women (1.61 ng/mL and 0.43 ng/mL, *p* = 0.004, respectively). The levels of TE in non-endometriosis patients (1.54 ng/mL) were also higher than in the healthy subjects; however, the differences were insignificant. To the best of our knowledge, this study is the first in which the serum concentration of telomerase was analyzed in a context of endometriosis. Some authors reported that blood plasma or serum telomerase activity is a good oncomarker; however, it is non-specific [56]. Endometriosis is a benign disease, but due to some features such as angiogenesis, tissue invasion and metastasis, it can be classified as a borderline malignant disease [57,58]. Nevertheless, Sofiyeva et al. [59] concluded that telomerase activity is useless as a biomarker in peripheral blood analysis for endometriosis. The authors explained this fact with the benign character of endometriosis and undetectably low levels of endometriotic cells in peripheral blood in comparison to malignant conditions [59].

The serum concentrations of advanced oxidation protein products were significantly higher in endometriosis and non-endometriosis than in healthy women (mean values: 235.09 µmol/L, *p* < 0.001, 196.27 µmol/L, *p* < 0.028 and 105.16 µmol/L, respectively). Jana et al. [38] also observed that serum AOPP concentrations were significantly increased in endometriosis women as compared with the control group (155.1 µmol/L vs. 90.60 µmol/L, *p* ≤ 0.001). It should be mentioned that in the discussed study, the control group was composed of women with tubal factor infertility. The authors concluded that increased serum levels of AOPP in endometriosis are in agreement with increased oxidative stress reported in these women [38]. AOPP was taken into consideration as an important inflammatory mediator in various chronic diseases [29], but in our study, we demonstrated the correlations only with CA125 concentrations (r = 0.335, *p* < 0.005) among other inflammatory parameters previously verified by us [32] (Supplementary Materials, Table S1). As it turns out, the higher serum AOPP levels were found even in the early stages of endometriosis (stage I and II) in comparison to patients without endometriosis (106.2 µmol/L and 100.9 µmol/L, respectively), but they were insignificant [36].

The role of AOPP in disease prognosis is becoming increasingly important, mainly due to the predictability of the severity of oxidative stress, but also due to the technical aspects such as sensitivity, stability, easy determination and, of course, low cost [29]. Studies on rat model published last year by Liu et al. [60] showed that AOPP accumulation can promote the proliferation and migration of endometrial epithelial cells via ERK and P38 signal, both in vivo and in vitro. Additionally, after the use of antioxidants or inhibitors of ERK and P38, the negative effect of AOPP on endometrial epithelial cells was attenuated [60]. These findings could be of great importance, as not only do they put a new insight into how biomarkers influence endometrial epithelial cells, but they also help us to understand the pathogenesis of endometrial diseases, including endometriosis.

In the ROC curve analysis, we selected three parameters as possible endometriosis markers: FRAP, telomerase and AOPP and, according to AUC, all of them have a moderate clinical value (0.704, 0.784 and 0.822, respectively). The sensitivity and specificity of these parameters were, respectively: 0.833, 0.588 (cut off point: 0.95 mmol/L); 0.944, 0.556 (cut off point: 0.26 ng/mL); and 0.706, 0.824 (cut off point: 152.82 µmol/L). The significance of these parameters was also confirmed in the logistic regression analysis (Supplementary Materials, Tables S1 and S2). The results of the aforementioned analysis corresponded with the hypothesis that oxidative stress plays an important role in the pathophysiology of endometriosis, especially in the disease association with a widespread inflammatory response [5]. This mechanism is visible systemically, because, in the sera of patients with endometriosis, higher total oxidant status and oxidative stress index were observed [37]. It has been also suggested that oxidative stress in patients with endometriosis may be responsible for the aggressiveness of the disease [18]. Amreen et al. [61] confirmed this hypothesis, and they showed the associations between increasing serum levels of oxidative stress parameters with increase in the severity of the disease.

It should be pointed that serum parameters of oxidative-antioxidant balance may represent oxidative stress due to other diseases, which may accompany endometriosis. This is why researchers prefer the measurements in peritoneal fluid over measurements in serum [5]. However, in our study, we aimed to verify the diagnostic utility of oxidative stress markers associated with endometriosis and the advisability of performing the determinations in an easily available material which is blood serum. Moreover, we would like to emphasize (as we have in our previous study [32]), how important it is to select an appropriate control group, because, in most publications, this group consists of women with mild gynecological disorders and without endometriosis. Our work evaluates the results obtained for patients with endometriosis and those with mild gynecological disorders, in comparison to the group of healthy, premenopausal women who were non-pregnant and who were without any gynecological problems, illness symptoms and disease history connected with endometriosis. The results of the present study may also suggest that the use of antioxidant therapy may be important in the treatment of endometriosis, reducing the negative effects of the disease; however, these findings should be verified also in terms of checking which antioxidants may be used to obtain a therapeutic effect.

### Strengths and Limitations

Strengths:

1. To our knowledge, the concentrations of serum SIRT3, SIRT5, SIRT6 and telomerase in patients with endometriosis were analyzed for the first time.
2. The three blood serum oxidative stress markers selected by us, FRAP, telomerase and AOPP, seem to be the most promising parameters for advanced endometriosis detection.
3. The analysis of 11 oxidative stress biomarkers’ expression and the determination of their importance in endometriosis are key to understanding the mechanisms involved in the development of the disease.
4. The results obtained for the endometriosis group were compared to those determined for healthy women who were our control group, which is more appropriate from the point of view of drawing constructive conclusions than comparing to a group of patients without endometriosis but suffering from other gynecological diseases.

Limitations:

1. Serum oxidative stress biomarkers may reflect an oxidative state that may be caused by diseases other than endometriosis.
2. Some parameters of oxidative stress are not stable and may fluctuate under the influence of many factors, including hormones and the day of the menstrual cycle.
3. Limitations are also due to the relatively small group size and the collection of venous blood samples regardless of the day of the menstrual cycle.

## 5. Conclusions

We verified the diagnostic utility of oxidative stress markers, determined in an easily available material—blood serum, which is associated with endometriosis development. Proposing the parameters usable in non-invasive endometriosis diagnostics was also a priority for us. The serum oxidative stress parameters FRAP, TE and AOPP seem to be the promising markers of oxidative-antioxidant balance, differentiating endometriosis patients from healthy women without any gynecological and inflammatory disorders; however, all parameters examined were comparable in the groups of advanced endometriosis and non-endometriosis, and the observed differences were insignificant. Taking the above into consideration, we would like to once again underline that the selection of an appropriate control group of healthy women without any gynecological problems, illness symptoms and disease history connected with endometriosis is especially important. Currently, there are very limited data examined on the associations between endometriosis development and sirtuins expression. To date, and to the best of our knowledge, there were no studies analyzing the concentrations of serum sirtuins 3, 5, 6 and telomerase in patients with endometriosis, which adds value to our research. We showed our results as novel findings on the possible role of the sirtuins family in endometriosis. The findings of the present study could be of great importance as they not only help to understand the pathogenesis of endometrial diseases, including endometriosis, but they also put a new insight into how biomarkers of oxidative stress influence endometrial epithelial cells and also may direct the future studies on this topic. Additionally, our results suggest that the use of antioxidant therapy may be important in the endometriosis treatment, which is worth considering, also taking into account the selection of the appropriate antioxidant that may be used to obtain a therapeutic effect.

## Figures and Tables

**Table 1:** The general characteristics of study groups.

	E n = 43	NE n = 35	Control n = 18
	Median (IQR)	Median (IQR)	Median (IQR)
BMI [kg/m^2^]	24 (22–26)	26 (24–28)	24 (21–26)
Age [years]	34 (30–41)	40 (33–42)	39 (35–41)
Reason for surgery [n, %]	Moderate stage (III E) 20, 46.5% Severe stage (IV E) 23, 53.5%	Leiomyoma 13, 37.0% Benign ovarian cyst 3, 8.5% Cervical intraepithelial neoplasia grade 3 12, 34.0% Ovarian teratoma 3, 8.5% Uterine polyp 1, 3.0% Borderline cystadenoma mucinosum 1, 3.0% BRCA1 mutation 2, 6.0%	Not applicable

E—endometriosis group; III E—moderate group of endometriosis (stage III according to rAFS classification); IV E—severe group of endometriosis (stage IV according to rAFS classification); NE—non-endometriosis group; IQR—interquartile range with values between quartile 1 (Q1) and quartile 3 (Q3); n—number of participants. Based on our previous work [32].

**Table 2:** The concentration values of oxidative-antioxidant balance parameters.

	E n = 43	III E n = 20	IV E n = 23	NE n = 35	Control n = 18
	Mean ± SD	Mean ± SD	Mean ± SD	Mean ± SD	Mean ± SD
TAC					
TAS [mmol/L]	1.52 ± 0.23	1.53 ± 0.19	1.52 ± 0.27	1.61 ± 0.25	1.60 ± 0.22
FRAP [mmol/L]	1.08 ± 0.22 *p* = 0.015 *	1.09 ± 0.27	1.07 ± 0.18 *p* = 0.017 *	1.14 ± 0.28 *p* = 0.017 *	0.95 ± 0.22
Low-molecular-weight antioxidants					
ALBUMIN [g/dL]	4.45 ± 0.66	4.68 ± 0.66	4.25 ± 0.60	4.29 ± 0.49	4.26 ± 0.19
T-BIL [mg/dL]	0.56 ± 0.33	0.54 ± 0.24	0.58 ± 0.40	0.68 ± 0.41	0.59 ± 0.24
URIC ACID [mg/dL]	4.78 ± 0.91	4.86 ± 1.13	4.72 ± 0.68	4.75 ± 1.40	4.72 ± 0.95
Iron					
IRON [μg/dL]	101.74 ± 62.80	122.70 ± 63.17	83.52 ± 57.76 *p* = 0.025 * p = 0.013 **	111.91 ± 76.12	105.89 ± 38.24
SIRTs and TE					
SIRT3 [ng/mL]	12.09 ± 7.06	12.30 ± 6.84	11.94 ± 7.40	11.00 ± 6.23	10.74 ± 6.11
SIRT5 [ng/mL]	8.09 ± 4.38	8.39 ± 4.34	7.83 ± 4.54	9.46 ± 5.45	7.98 ± 5.98
SIRT6 [ng/mL]	12.32 ± 16.84	8.51 ± 15.90	14.76 ± 17.71	14.54 ± 17.66	7.17 ± 10.40
TE [ng/mL]	1.61 ± 1.88 *p* = 0.004 *	1.01 ± 0.60 *p* = 0.021 *	2.00 ± 2.32 *p* = 0.017 *	1.54 ± 2.75	0.43 ± 0.52
AOPP					
AOPP [µmol/L]	235.09 ± 143.11 *p* < 0.001 *	225.63 ± 156.64 *p* = 0.005 *	242.55 ± 135.40 *p* < 0.001 *	196.27 ± 180.94 *p* = 0.028 *	105.16 ± 49.24

A two-tailed *p*-value of less than 0.05 was considered significant. Significant differences versus: * control group of healthy women, ** stage III of endometriosis. E—endometriosis group; III E—moderate group of endometriosis (stage III according to rAFS classification); IV E—severe group of endometriosis (stage IV according to rAFS classification); NE—non-endometriosis group; AOPP—advanced protein oxidation products; FRAP—ferric reducing antioxidant power; SIRT3—sirtuin 3; SIRT5—sirtuin 5; SIRT6—sirtuin 6; TAC—total antioxidant capacity; TAS—total antioxidant status; TE—telomerase; T-BIL—total bilirubin.

**Table 3:** The correlations between concentrations of determined parameters.

Parameter	TAS	FRAP	ALB	T-BIL	UA	IRON	SIRT3	SIRT5	SIRT6	TE
FRAP	r = 0.576 *p* < 0.001									
ALB	r = −0.180 *p* = 0.038	r = −0.339 *p* < 0.001								
T-BIL	NS	NS	r = 0.393 *p* < 0.001							
UA	r = 0.264 *p =* 0.002	r = 0.590 *p* < 0.001	NS	NS						
IRON	NS	NS	r = 0.344 *p <* 0.001	r = 0.405 *p* < 0.001	r = 0.176 *p* = 0.039					
SIRT3	NS	r = −0.305 *p* < 0.001	r = 0.298 *p* < 0.001	NS	NS	r = 0.184 *p* = 0.043				
SIRT5	NS	r = −0.229 *p* = 0.019	NS	NS	r = −0.199 *p* = 0.041	r = 0.192 *p* = 0.050	r = 0.845 *p* < 0.001			
SIRT6	NS	NS	NS	NS	r = −0.233 *p* = 0.042	NS	r = 0.770 *p* < 0.001	r = 0.863 *p* < 0.001		
TE	NS	r = 0.431 *p* < 0.001	r = −0.323 *p* = 0.004	NS	r = 0.356 *p* = 0.001	NS	r = −0.227 *p* = 0.049	NS	NS	
AOPP	r = −0.211 *p* = 0.026	NS	NS	NS	r = 0.378 *p <* 0.001	r = 0.235 *p* = 0.012	NS	NS	NS	r = 0.363 *p* = 0.001

Spearman’s rank correlation was used to check the correlations between analyzed parameters, and a *p* value of less than 0.05 was considered significant; NS—not significant. AOPP—advanced protein oxidation products; ALB—albumin; FRAP—ferric reducing antioxidant power; SIRT-3—sirtuin 3; SIRT-5—sirtuin 5; SIRT-6—sirtuin 6; TAS—total antioxidant status; TE—telomerase; T-BIL—total bilirubin; UA—uric acid.

## Data Availability

The data presented in this study are available upon reasonable request from the corresponding author.

## Supplementary Materials

The following are available online at https://www.mdpi.com/article/10.3390/antiox10071097/s1, Table S1: The results of univariate logistic regression models; Table S2: Backward stepwise regression after verification of the collinearity of the predictors (parameters); Table S3: The correlations between concentrations of serum parameters of inflammation and oxidative-antioxidant balance.
