# Supplementary material for: Is There a Balance in Oxidative-Antioxidant Status in Blood Serum of Patients with Advanced Endometriosis?
Source: Antioxidants (Basel). 2021 Jul 8;10(7):1097. doi: 10.3390/antiox10071097 (PMC8301022; doi:10.3390/antiox10071097)
Supplement: Supplementary file 1 [file antioxidants-10-01097-s001.zip › antioxidants-1248709-supplementary.pdf]

Table S1. The results of univariate logistic regression models.

Parameter / Analyzed effect	TAS		FRAP		ALB		T-BIL		UA		IRON		SIRT3		SIRT5		SIRT6		TE		AOPP	
	OR (95%)	*p*-value	OR (95%)	*p*-value	OR (95%)	*p*-value	OR (95%)	*p*-value	OR (95%)	*p*-value	OR (95%)	*p*-value	OR (95%)	*p*-value	OR (95%)	*p*-value	OR (95%)	*p*-value	OR (95%)	*p*-value	OR (95%)	*p*-value
**E vs.** C	0.241 (0.022 – 2.598)	0.241	24.744 (0.859 – 712.600)	0.061	1.915 (0.660 – 5.560)	0.232	0.721 (0.124 – 4.187)	0.716	1.091 (0.586 – 2.032)	0.784	0.999 (0.989 – 1.008)	0.999	1.033 (0.943 – 1.132)	0.483	1.004 (0.891 – 1.132)	0.943	1.029 (0.978 – 1.083)	0.277	4.633 (1.013-21.187)	0.048	1.018 (1.005 – 1.030)	0.005
**III E vs.** C	0.184 (0.007 – 4.735)	0.307	11.718 (0.522 – 263.161)	0.121	6.873 (1.183 – 39.923)	0.032	0.398 (0.026 – 6.084)	0.508	1.154 (0.611 – 2.180)	0.659	1.007 (0.993 – 1.020)	0.330	1.040 (0.933 – 1.161)	0.478	1.015 (0.887 – 1.162)	0.826	1.010 (0.939 – 1.085)	0.797	5.893 (1.036–33.527)	0.046	1.015 (1.002 – 1.027)	0.026
**IV E vs.** C	0.228 (0.015 – 3.387)	0.283	30.593 (0.670 – 1397.438)	0.079	0.943 (0.238 – 3.739)	0.933	0.887 (0.138 – 5.719)	0.900	1.003 (0.458 – 2.200)	0.993	0.991 (0.978 – 1.004)	0.168	1.028 (0.932 – 1.134)	0.578	0.994 (0.873 – 1.132)	0.929	1.042 (0.983 – 1.104)	0.165	2.984 (0.711–12.523)	0.135	1.021 (1.006 – 1.036)	0.006
**E vs.** NE	0.211 (0.030 – 1.499)	0.120	0.383 (0.061 – 2.397)	0.305	1.622 (0.731 – 3.600)	0.234	0.381 (0.103 – 1.406)	0.148	1.026 (0.693 – 1.519)	0.897	0.998 (0.991 – 1.004)	0.515	1.026 (0.951 – 1.105)	0.509	0.943 (0.847 – 1.051)	0.288	0.992 (0.956 – 1.030)	0.678	1.012 (0.779–1.315)	0.927	1.002 (0.998 – 1.005)	0.352
**III E vs.** NE	6.630 (0.121 – 2.676_	0.229	0.478 (0.058 – 3.967)	0.494	3.297 (1.159 – 9.378)	0.025	0.242 (0.034 – 1.733)	0.158	1.069 (0.700 – 1.631)	0.758	1.002 (0.994 – 1.010)	0.587	1.032 (0.938 – 1.135)	0.515	0.957 (0.837 – 1.093)	0.515	0.975 (0.917 – 1.037)	0.421	0.875 (0.517–1.481)	0.619	1.001 (0.997 – 1.005)	0.593
**IV E vs.** NE	0.202 (0.020 – 2.011)	0.173	0.313 (0.030-3.265)	0.332	0.862 (0.309 – 2.405)	0.777	0.480 (0.110 – 2.098)	0.329	0.975 (0.616 – 1.542)	0.912	0.994 (0.986 – 1.002)	0.136	1.021 (0.939 – 1.112)	0.623	0.935 (0.821 – 1.065)	0.310	1.001 (0.960 – 1.043)	0.973	1.068 (0.816–1.398)	0.633	1.002 (0.998 – 1.005)	0.350
**E vs.** C+NE	0.201 (0.032 – 1.277)	0.089	1.032 (0.202 – 5.724)	0.970	1.840 (0.842 – 4.020)	0.126	0.434 (0.121 – 1.559)	0.200	1.038 (0.721 – 1.495)	0.840	0.998 (0.992 – 1.004)	0.535	1.029 (0.962 – 1.099)	0.407	0.969 (0.883 – 1.064)	0.511	1.004 (0.969 – 1.040)	0.816	1.129 (0.872–1.461)	0.357	1.004 (1.000 – 1.007)	0.043

Significant differences (*p*-value of less than 0.05) are shown in red bold. **C –** control group of healthy women, **E** - endometriosis group, **III E**- group of moderate endometriosis (stage III according to rAFS classification), **IV E** –group of severe endometriosis (stage IV according to rAFS classification), **NE** - non-endometriosis group, **OR -** Odds Ratio.

Table S2. Backward stepwise regression after verification of the collinearity of the predictors (parameters).

Parameters	Odds Ratio (OR)	95% Confidence Interval (CI)	*p*-value
		**E vs.** C	
TAS	0.001	0.000 – 0.651	0.037
FRAP	83534970.264	9.901 – 704818378400096.000	0.025
AOPP	1.017	1.002 – 1.032	0.028
UA	0.048	0.003 – 0.821	0.036
		**III E vs.** C	
FRAP	37.681	1.112 – 1265.890	0.043
		**IV E vs.** C	
Alb	0.001	0.000 – 0.743	0.040
SIRT6	1.132	0.999 – 1.282	0.052
AOPP	1.032	1.001 – 1.065	0.041
		**E vs.** NE	
T–BIL	0.043	0.002 – 0.781	0.033
		**III E vs.** NE	
		**NS**	
		**IV E vs.** NE	
AOPP	1.009	1.001 – 1.017	0.026
		**E vs.** C+NE	
AOPP	1.009	1.002 – 1.016	0.014
T-BIL	0.051	0.003 – 0.955	0.047

Significant differences (*p*-value of less than 0.05) are shown in red bold. **C** – control group of healthy women, E - endometriosis group, III E- group of moderate endometriosis (stage III according to rAFS classification), IV E –group of severe endometriosis (stage IV according to rAFS classification), NE - non-endometriosis group, OR - Odds Ratio.

Table S3. The correlations between concentrations of serum parameters of inflammation and oxidative-antioxidant balance.

Parameter	BMI	CA 125	Estradiol	PRL	IgG	hs-CRP	IL-1β	IL-6	YKL-40
**TAS**	r=-0.214 *p*=0.011	NS	NS	NS	NS	NS	NS	r=0.223 *p*=0.008	NS
**FRAP**	NS	NS	NS	r=0.221 *p*=0.012	r=-0.350 *p*<0.001	r=0.399 *p*<0.001	r=0.380 *p*<0.001	r=0.386 *p*<0.001	NS
**ALB**	NS	NS	r=0.437 *p*<0.001	r=-0.183 *p*=0.036	r=0.594 *p*<0.001	r=-0.553 *p*<0.001	r=-0.232 *p*=0.006	r=-0.453 *p*<0.001	NS
**T-BIL**	NS	NS	r=0.249 *p*=0.004	NS	r=0.337 *p*<0.001	r=-0.293 *p*<0.001	NS	r=-0.175 *p*=0.041	NS
**UA**	NS	NS	NS	NS	NS	NS	r=0.270 *p*=0.001	NS	NS
**IRON**	NS	r=-0.294 *p*<0.001	r=0.217 *p*=0.012	NS	r=0.338 *p*<0.001	r=-0.540 *p*<0.001	NS	r=-0.438 *p*<0.001	NS
**SIRT3**	NS	NS	r=0.268 *p*=0.003	NS	r=0.400 *p*<0.001	r=-0.239 *p*=0.008	NS	r=-0.184 *p*=0.043	r=0.743 *p*<0.001
**SIRT5**	NS	NS	NS	NS	r=0.275 *p*=0.004	NS	r=0.270 *p*=0.005	NS	r=0.798 p<0.001
**SIRT6**	NS	NS	NS	NS	r=0.244 *p*=0.032	NS	r=0.399 *p*<0.001	NS	r=0.829 *p*<0.001
**TE**	NS	r=0.262 *p*=0.027	NS	r=0.314 *p*=0.005	r=-0.275 *p*=0.015	r=0.333 *p*=0.003	r=0.396 *p*<0.001	r=0.300 *p*=0.008	NS
**AOPP**	NS	r=0.335 *p*<0.001	NS	NS	NS	NS	NS	NS	NS

The parameters of oxidative-antioxidant balance were correlated with inflammatory parameters determined by us previously (Kokot et al. [47]). AOPP - advanced protein oxidation products, ALB - albumin, CA-125 - cancer antigen 125, FRAP - ferric reducing antioxidant power, hs-CRP – high sensitive C-reactive protein, IgG – immunoglobulin G, IL-1β – interleukin 1β, IL-6 – interleukin 6, PRL – prolactin, SIRT-3 - sirtuin 3, SIRT-5 - sirtuin 5, SIRT-6 - sirtuin 6, TAS - total antioxidant status, TE – telomerase, T-BIL - total bilirubin, UA - uric acid, YKL-40 - chitinase-3-like protein 1.
